# Multi-Strategy Enhanced White Shark Optimizer for Solving Job Shop Scheduling Problem

**DOI:** 10.3390/biomimetics11060372

**Published:** 2026-05-27

**Authors:** Li Cao, Meng Li, Ken Chen, Yinggao Yue, Yang Qiu, Zihao Cheng

**Affiliations:** 1 School of Electronics and Electrical Engineering, Wenzhou University of Technology, Wenzhou 325035, China; 2 Zhejiang Quality Inspection Center of High and Low-Voltage Electrical Products, Yueqing 325603, China; 3 College of Control Science and Engineering, Zhejiang University, Hangzhou 310027, China

**Keywords:** white shark optimizer, job shop scheduling problem, tent chaotic map, Levy flight, elite opposition-based learning, convergence accuracy

## Abstract

Aiming at the inherent limitations of the basic White Shark Optimizer (WSO), such as insufficient population diversity, unbalanced global and local search mechanisms, and weak convergence in the later stage, this paper proposes an Improved White Shark Optimizer (IWSO). The algorithm is improved from the following three aspects: Firstly, the Tent chaotic map is introduced to replace the traditional random initialization in the population initialization stage. Secondly, an adaptive nonlinear convergence factor and a dynamic inertia weight adjustment strategy are designed to focus on the fine search in the neighborhood of the optimal solution. Thirdly, the Levy flight perturbation mechanism and the elite opposition-based learning strategy are integrated to expand the search range and further accelerate the convergence speed. To verify the effectiveness and superiority of the IWSO algorithm, the CEC2017 test suite is selected for simulation experiments, and the IWSO is systematically compared with seven other representative swarm intelligence algorithms. The experimental results show that the IWSO is significantly superior to all comparison algorithms in multiple evaluation indicators, including minimum makespan, average convergence value, standard deviation, and successful convergence rate, on scheduling instances of different scales and difficulties. The convergence curve remains leading throughout the iteration process and shows a smoother convergence trend. The multi-strategy enhanced white shark optimizer proposed in this paper effectively overcomes the inherent defects of the basic algorithm, significantly improves the solution accuracy and convergence efficiency of the job shop scheduling problem, and has high theoretical research value and practical engineering application prospects. In the future, the multi-strategy improved White Shark Optimizer will be extended to multi-objective job shop scheduling, dynamic disturbance job shop scheduling, and large-scale production scheduling scenarios with numerous workpieces and machines.

## 1. Introduction

With the rapid development of intelligent manufacturing and flexible production, the Job Shop Scheduling Problem (JSP) has become a core link restricting the improvement of production efficiency [1,2]. As a typical NP-hard combinatorial optimization problem, JSP is characterized by high dimensionality, multiple constraints, strong coupling, and multi-modality [3]. The feasible solution space increases exponentially with the growth of the number of jobs and machines, making it difficult for traditional exact algorithms to obtain satisfactory solutions within a reasonable time [4,5]. Meta-heuristic intelligent optimization algorithms have become the mainstream method for solving job shop scheduling problems due to their advantages of gradient-free information, strong global search capability, and wide adaptability [6]. The Job Shop Scheduling Problem (JSP) is one of the most classic and representative combinatorial optimization problems in the manufacturing field. It mainly studies how to reasonably arrange the processing sequence of multiple jobs on limited machine resources to optimize production indicators such as maximum makespan, total tardiness, and machine utilization [7,8]. With the rapid development of global manufacturing towards intelligence and flexibility, enterprises have put forward higher requirements for the real-time performance, robustness, and global optimality of production scheduling [9,10]. However, JSP is essentially high-dimensional, multi-constrained, strongly nonlinear, and multi-modal, belonging to the NP-hard problem [11,12]. When the number of jobs and machines increases, the number of feasible solutions grows exponentially, and traditional mathematical programming methods (such as integer programming and branch-and-bound method) find it difficult to obtain satisfactory solutions within a reasonable time [13,14,15].

As a meta-heuristic method simulating the behavior of biological groups in nature, swarm intelligence optimization algorithms have been widely used to solve various complex optimization problems owing to their gradient-free information, strong parallel search capability, and easy implementation [16,17]. Classical algorithms such as Particle Swarm Optimization [18], Ant Colony Optimization [19], Whale Optimization Algorithm [20], and Harris Hawks Optimization [21] have achieved numerous research results in the field of job shop scheduling. In the field of metaheuristic algorithm research, the No Free Lunch theorem (NFL) is the fundamental theory guiding algorithm design and performance evaluation. This theorem explicitly states that there is no universal algorithm that can maintain optimal performance on all types of optimization problems, and each optimization algorithm has specific applicable scenarios and performance boundaries. When performing well on one type of problem, there are often performance shortcomings on another type of problem. Constrained by the NFL theorem, existing classical swarm intelligence algorithms are difficult to simultaneously adapt to complex scenarios such as continuous benchmark optimization, engineering constraint design, and discrete workshop scheduling. Nevertheless, single swarm intelligence algorithms still commonly suffer from slow convergence, easy trapping in local optima, and insufficient exploitation ability in the later stage when dealing with large-scale and multi-constrained job shop scheduling problems [22,23]. Therefore, it is of great theoretical value and practical significance to improve existing algorithms with multi-strategy enhancements and balance their global exploration and local exploitation capabilities [24].

The White Shark Optimizer (WSO) is a novel meta-heuristic algorithm proposed in recent years [25]. It realizes optimization search by simulating the exploration, tracking, and attacking behaviors of white sharks during hunting. With a simple structure and few parameters, it performs well in some continuous optimization problems [26,27]. Firstly, we add the comparative analysis with the existing improved variants of WSO, focusing on the comparison with the Tent map-based improved WSO algorithm in reference [27]. We conduct horizontal comparison from the dimensions of initialization strategy, search mechanism, fusion strategy, test scenario, convergence accuracy and robustness, and illustrate the advantages of the proposed multi-strategy fusion improved WSO in this paper compared with the single Tent map improved WSO, including stronger balance ability of global exploration and local exploitation, more perfect mechanism of jumping out of local optimum, and better adaptation to discrete optimization scenarios of job shop scheduling. Meanwhile, we supplement the numerical result comparison and performance difference analysis. Secondly, we add the adaptability comparison discussion between meta-heuristic algorithms and Deep Reinforcement Learning (DRL). The CEC2017 continuous optimization, piston rod engineering constrained optimization, and job shop scheduling NP-hard discrete optimization problems tested in this paper have the characteristics of complex solution space, no gradient information, multiple constraints, and solvable with small-scale samples. Meta-heuristic algorithms do not require training samples and model prior knowledge, with a simple structure, convenient parameter tuning, and feasible optimal solutions can be obtained in a single operation, which are suitable for small and medium-scale combinatorial optimization and engineering optimization problems. In contrast, Deep Reinforcement Learning requires a large amount of training data, long network training time, complex hyperparameter tuning, and its generalization depends on training scenarios. The solution efficiency and convenience are much lower than meta-heuristic algorithms in the small-scale benchmark instances and engineering scheduling problems in this paper, which clarifies the adaptation advantages of meta-heuristic algorithms. However, the basic WSO still has obvious defects in population initialization, search mechanism balance, and escaping local optima, which limit its application effect in discrete combinatorial optimization problems (such as job shop scheduling) [28]. To this end, this paper proposes an Improved White Shark Optimizer (IWSO). By introducing the Tent chaotic map, adaptive nonlinear convergence factor, dynamic inertia weight, Levy flight perturbation, and elite opposition-based learning, the global search capability, convergence accuracy, and robustness of the algorithm are comprehensively improved [29]. The algorithm is applied to solve the job shop scheduling problem, aiming to provide an efficient and reliable optimization method for production scheduling under the background of intelligent manufacturing [30].

Aiming at the shortcomings of the basic White Shark Optimizer (WSO), such as insufficient population diversity, unbalanced global and local search, and easy trapping in local optima in the later stage, this study proposes a multi-strategy enhanced white shark optimizer (IWSO) by integrating Tent chaotic map, adaptive nonlinear convergence factor, dynamic inertia weight, Levy flight perturbation and elite opposition-based learning, and applies it to the job shop scheduling problem. The main contributions of this paper are as follows:

1. The Tent chaotic map is introduced for population initialization to replace the traditional random initialization method, which improves the ergodic uniformity of the initial population and reduces the risk of premature convergence from the source.
2. A collaborative adjustment strategy of adaptive nonlinear convergence factor and dynamic inertia weight is designed to dynamically balance the global exploration and local exploitation capabilities of the algorithm, enabling wide-area search in the early iteration and fine optimization in the later stage, thus improving convergence accuracy and stability.
3. The Levy flight perturbation and elite opposition-based learning strategy are integrated to trigger large-span search and expand the solution space when the algorithm stagnates, which effectively enhances the ability to jump out of local optima and accelerates the overall convergence speed.
4. The results show that IWSO is significantly superior to many mainstream intelligent algorithms in solution accuracy, convergence speed, and robustness, and has good engineering applicability.

## 2. Mathematical Model of the Job Shop Scheduling Problem

The classical job shop scheduling problem can be described as follows: there are *n* jobs to be processed on *m* machines [31]. Each job contains several operations, each of which is assigned to a specific machine, and the processing time is known in advance. The processing sequence of all jobs on each machine is predetermined, while no sequential constraints exist among operations of different jobs [32]. The goal of scheduling optimization is to determine the processing sequence and start–end time of each operation on each machine such that a certain performance index is optimized under all process constraints and resource constraints [33].

To facilitate mathematical modeling, the following basic assumptions are usually made: all jobs are available for processing at time zero, each machine can process only one operation at a time, each job can be processed by only one machine at a time, once an operation starts processing, it cannot be interrupted (non-preemptive); the processing sequence of operations for the same job must follow the process route. There is no buffer limit between machines, meaning that an operation can wait for the next machine to become idle after completion, the assumption of no buffer limit between machines in this paper is the standard simplified assumption of classic job shop scheduling benchmark instances, which is used to strip external interference factors such as material transfer, loading and unloading equipment and buffer capacity, and focus on the core process sequencing and machine processing timing optimization. Uncertain factors such as machine breakdown and job transportation time are neglected [34].

For the convenience of mathematical modeling, the following basic assumptions are usually made, and the symbols and variables are shown in Table 1.

The constraints of workshop scheduling problems mainly include process sequence constraints and machine resource constraints.

(1) Process sequence constraint: For adjacent processes of the same workpiece, the next process can only begin after the previous process is completed. If there is a sequential relationship between the processes *O_ij_* and *O_ik_* of workpiece *i*, then:

(1) $$S_{ik}\geq C_{ij}=S_{ij}+p_{ij}$$

(2) Machine resource constraint: Each machine can only process one process at a time. For two different processes *O_ij_* and *O_hj_* processed on the same machine *jj*, the processing sequence must be determined. If *O_ij_* is processed before *O_hj_*, then:

(2) $$S_{hj}\geq C_{ij}=S_{ij}+p_{ij}$$

On the contrary, if *O_hj_* is processed before *O_ij_*, then:

(3) $$S_{ij}\geq C_{hj}=S_{hj}+p_{hj}$$

The above relationship can be uniformly represented by the decision variable $x_{ij}^{hj}$ as follows:

(4) $$S_{hj}+M\cdot \left(1-x_{ij}^{hj}\right)\geq S_{ij}+p_{ij}$$

(5) $$S_{ij}+M\cdot x_{ij}^{hj}\geq S_{hj}+p_{hj}$$

Among them, *M* is a sufficiently large positive number.

(3) Non negative constraint on processing time:

(6) $$S_{ij}\geq 0,p_{ij}>0\;\;\;\;$$

The maximum completion time *C_max_* is the most commonly used optimization objective in workshop scheduling problems, which reflects the length of the production cycle and directly affects the inventory of work in progress and production efficiency. The objective function with the goal of minimizing the maximum completion time can be expressed as:

(7) $$minC_{max}=\min\left(\underset{1\leq i\leq n}{\max}\;C_{i}\right)$$

Among them is *C_i_* = max*C_ij_* (1 ≤ *j* ≤ *m*), which is the completion time of the last process of workpiece *ii*.

In addition to the maximum completion time, other goals may also be considered in actual production, such as minimizing the total completion time (Total Completion Time): $\sum_{i=1}^{n}C_{i}$. Maximum Latency: $max\left(0,Ci-di\right)$, where *d_i_* is the delivery time. Minimize Total Tardiness: $\sum max\left(0,Ci-di\right)$. Minimize machine idle time or energy consumption, etc. This article aims to minimize the maximum completion time as the optimization objective.

## 3. White Shark Optimizer (WSO)

The White Shark Optimizer (WSO) is a novel meta-heuristic optimization algorithm proposed by Malik Braik in 2022. It corely simulates the behavior of white sharks´ tracking, encircling, and preying on prey by virtue of hearing, smell, and vision, with both global exploration and local exploitation capabilities [35,36]. It features a simple structure and few parameters. The algorithm simulates the process of white sharks locking and tracking prey through hearing and smell, and cooperatively encircling prey through group behavior [37]. The optimization search is divided into two core stages: sensory tracking (global exploration) and group encirclement (local exploitation). Optimization is realized through a velocity update and dual-strategy position update [38].

(1) Individual position and velocity definition

Assuming the *d*-dimensional position vector of the *i*-th great white shark in the population is:

(8) $$\begin{array}{c} W^{i}=(w^{i1},w^{i2},\cdots ,w^{id}) \end{array}$$

In the formula, $w^{id}$ represents the *d*-dimensional position component of the *i*-th shark.

(2) Position update (hearing/smell movement)

The great white shark perceives prey and updates its position through hearing and smell, and switches between two strategies with a threshold of $m_{v}$. The position update formula is:

(9) $$\begin{array}{c} w_{k+1}^{i}=\{\begin{array}{cc} w_{k}^{i}\cdot \neg ⊕\omega_{0}+u\cdot a+l\cdot b, & \mathrm{rand}<m_{v}\\ w_{k}^{i}+v_{k}^{i}/f, & \mathrm{rand}\geq m_{v} \end{array} \end{array}$$

In the formula, $m_{v}$ represents the strategy selection threshold, which controls the switching between global/local search. The parameters *u* and *l* represent the upper and lower bounds of the d-th dimension of the search space. $\omega_{0}$ is the boundary dimension identifier, where $\neg ⊕\omega_{0}$ is its binary inversion. $\omega_{0}$ is the dimension boundary identification vector, which is used to mark whether the position dimension of the white shark individual exceeds the upper and lower bounds of the search space. ¬ represents the bitwise NOT operation in binary, which reverses the boundary identification vector through bit operation. ⊕ denotes the dimension-wise XOR operation to realize the position correction compensation of out-of-bounds dimensions. The parameter *a* represents the dimension components that exceed the upper bound, the parameter *b* represents the dimension components that are below the lower bound, and $v_{k}^{i}$ represents the velocity of the *i*-th shark in the *k*-th generation. The parameter *f* represents the wave frequency parameter (fixed constant). The parameter *rand* is a [0, 1] uniform random number [39,40].

(3) Strategy selection threshold

The threshold value $m_{v}$ is dynamically adjusted with iteration, and the calculation formula is:

(10) $$\begin{array}{c} m_{v}=\frac{1}{a_{0}+e^{(h/2-k)/a_{1}}} \end{array}\;$$

In the formula, the parameter *h* represents the maximum number of iterations. The parameter *k* represents the current iteration count. Parameters $a_{0}$ and $a_{1}$ represent the algorithm constant parameters.

(4) Velocity update formula

The speed update draws inspiration from the particle swarm optimization concept, combined with the contraction factor and learning coefficient, using the formula:

(11) $$\begin{array}{c} v_{k+1}^{i}=\mu [v_{k}^{i}+p_{1}(w_{\mathrm{gbest}}-w_{k}^{i})\cdot c_{1}+p_{2}(w_{\mathrm{ibest}}^{i}-w_{k}^{i})\cdot c_{2}] \end{array}$$

In the formula, the parameter *μ* represents the contraction factor, balancing the convergence of the algorithm. Parameters $p_{1}$ and $p_{2}$ represent the global optimal individuals and optimal learning weights. The parameter $w_{\mathrm{gbest}}$ represents the global optimal position of the population. $w_{\mathrm{ibest}}^{i}$ represents the historical optimal position of the *i*-th shark. $c_{1}$ and $c_{2}$ represent [0, 1] random numbers, adding search perturbations [41].

(5) Group behavior position update

The algorithm completes fish swarm capture in two modes, which are determined by the control factor $s_{s}$ to switch: the mode of approaching the optimal shark (${rand}_{3}<s_{s}$):

(12) $$\begin{array}{c} {\overset{˙}{w}}_{k+1}^{i}=w_{\mathrm{gbest}}+r_{1}\cdot D_{w}\cdot \mathrm{sgn}(r_{2}-0.5) \end{array}$$

Fish swarm cooperative disturbance mode (${rand}_{3}\geq s_{s}$):

(13) $$\begin{array}{c} w_{k+1}^{i}=\frac{w_{k}^{i}+{\overset{˙}{w}}_{k+1}^{i}}{2\times \mathrm{rand}} \end{array}$$

In the formula, $D_{w}=|w_{\mathrm{gbest}}-w_{k}^{i}|$ represents the distance between the shark and the global optimum. $r_{1}$, $r_{2}$, and $r_{3}$ are uniform random numbers [0, 1]. The parameter $s_{s}$ represents the mode switching control factor, and the calculation formula is: $\begin{array}{c} s_{s}=|1-e^{-a_{2}\cdot k/h}| \end{array}$. The parameter $a_{2}$ represents the algorithm constant, and *h* is the maximum number of iterations.

Although the original White Shark Optimizer (WSO) has the advantages of simple structure, few control parameters and flexible optimization framework by simulating the hunting behavior of great white sharks, and shows certain optimization potential in some continuous optimization problems, it still suffers from many inherent weaknesses and application limitations, making it difficult to directly solve complex high-dimensional optimization, multimodal complex landscape problems and NP-hard combinatorial optimization problems such as job shop scheduling. Firstly, the original WSO generates the initial population in a completely random manner, which lacks the ability of uniform ergodicity in the solution space. It is prone to individual aggregation and uneven distribution, resulting in insufficient initial search coverage and leaving hidden dangers of premature convergence from the early iteration stage. Secondly, there is no dynamic adjustment mechanism to balance global exploration and local exploitation in the original algorithm. The fixed strategy switching threshold and constant velocity updating weight cannot adaptively change with the iteration process, which easily leads to an imbalance of insufficient exploration in the early stage and excessive exploitation in the later stage, or vice versa, thus reducing the optimization efficiency. Thirdly, the original WSO lacks an effective population diversity maintenance mechanism. The similarity of population individuals becomes serious in the middle and later iterations. Once trapped in local optimal regions, the algorithm is difficult to jump out autonomously, resulting in obvious convergence stagnation. Meanwhile, the position update rule of the original algorithm contains ambiguous abstract operation symbols and vague boundary handling logic, and the neighborhood search lacks sufficient refinement, which leads to a significant decline in convergence accuracy when dealing with high-dimensional, multimodal, and strongly coupled complex optimization problems. In addition, the original WSO is designed for continuous space optimization without adapting to discrete encoding and process sequencing logic, so it cannot be directly applied to discrete combinatorial optimization problems such as job shop scheduling. In summary, the basic WSO exhibits obvious defects in population initialization quality, balance between global exploration and local exploitation, ability to escape local optima, accuracy of local neighborhood search, and adaptability to discrete scenarios. To address the above shortcomings and break through the performance bottlenecks of the original algorithm, this paper designs multiple improved strategies from the perspectives of population initialization optimization, adaptive search parameter adjustment, introduction of a global escape mechanism, and enhancement of local neighborhood search, and constructs a multi-strategy improved White Shark Optimizer (IWSO). The proposed IWSO effectively improves population diversity, global exploration capability, local exploitation accuracy, and adaptability to complex engineering optimization problems.

## 4. Multi-Strategy Enhanced White Shark Optimizer (IWSO)

The original WSO has obvious shortcomings in population initialization, balance between global and local search, and escaping local optima, making it difficult to be directly applied to discrete combinatorial optimization problems (such as job shop scheduling). Therefore, an Improved White Shark Optimizer (IWSO) is proposed. Three strategies are adopted to comprehensively improve the optimization performance of the algorithm:

(1) Tent chaotic map initialization

The chaotic map has the characteristics of ergodicity, non-repetition, and sensitivity to initial values, which can generate more uniform initial solutions than a random distribution [42]. Among various chaotic maps, the Tent map has better ergodic uniformity than the Logistic map and requires simple parameter settings [43]. This paper employs the Tent chaotic map for population initialization, with the specific form being:

$$y_{i+1}=2y_{i},\mathrm{when}\;0\leq y_{i}\leq 0.5.\;y_{i+1}=2\left(1-y_{i}\right),\;\mathrm{when}\;0.5<y_{i}\leq 1.$$

To avoid falling into fixed points and small cycles, random perturbations are introduced: $y_{i+1}=2y_{i}+rand\left(0,1\right)/N$, when $0\leq y_{i}\leq 0.5$.$y_{i+1}=2\left(1-y_{i}\right)+rand\left(0,1\right)/N$, when $0.5<y_{i}\leq 1$.

Where *N* is the population size, and *rand*(0, 1) is a uniformly random number within [0, 1]. After generating the chaotic sequence through the Tent map, it is mapped to the search space:

(14) $$X_{i,j}=lb_{j}+y_{i}\cdot \left(ub_{j}-lb_{j}\right)$$

This strategy makes the initial population distribution more uniform, increases the coverage of the understanding space, and thus reduces the risk of premature convergence [44].

(2) Adaptive nonlinear convergence factor and dynamic inertia weight

To balance global exploration and local exploitation capabilities, an adaptive nonlinear convergence factor *η*(*t*) and a dynamic inertia weight *w*(*t*) are introduced [45]. The convergence factor decays nonlinearly with the number of iterations, with a slow decay in the early stage to maintain strong global exploration ability, and a fast decay in the later stage to focus on local fine search [46]:

(15) $$\eta \left(t\right)=\eta_{max}-\lambda \cdot \left(\eta_{max}-\eta_{min}\right)\cdot \left(\frac{t}{T_{max}}\right)$$

where *t* is the current iteration count, *T_max_* is the maximum iteration count, *η_max_* = 0.9, *η_min_* = 0.2, and *λ* is the nonlinear adjustment factor (taken as *λ* = 2).

The inertia weight affects the degree to which the previous generation’s position affects the current update, using a cosine decreasing strategy

(16) $$w\left(t\right)=w_{min}+\left(w_{max}-w_{min}\right)\cdot \cos\left(\frac{\pi }{2}\cdot \frac{t}{T_{max}}\right)$$

Among them, *w_max_* = 0.9 and *w_min_* = 0.4. Early weight is relatively high, maintaining diversity in exploration. Reduce the weight in the later stage and enhance local development [47]. Firstly, *η_max_* = 0.9 and *η_min_* = 0.2 are the classic value intervals of convergence factors for meta-heuristic algorithms. We test the combined performance of the maximum value in the 0.8~1.0 interval and the minimum value in the 0.1~0.3 interval through multiple groups of comparative simulations, and verify that the combination of 0.9 and 0.2 can optimally balance the global exploration ability in the early iteration stage and local exploitation ability in the later iteration stage. Secondly, *w_max_* = 0.9 and *w_min_* = 0.4 are the classic values commonly used for dynamic inertia weight. After multiple rounds of simulation tests, this value can realize retaining population diversity with large weight in the early iteration stage and strengthening local fine search with small weight in the later iteration stage.

The convergence factor and inertia weight are integrated into the WSO position update, and the improved global exploration formula is:

(17) $$X_{i}^{t+1}=w\left(t\right)\cdot X_{i}^{t}+\eta \left(t\right)\cdot \left[r_{1}\cdot \left(X_{best}^{t}-X_{i}^{t}\right)+r_{2}\cdot \left(X_{rand}^{t}-X_{i}^{t}\right)\right]$$

Among them, *X_randt_* is a randomly selected individual position. The formula for local development is:

(18) $$X_{i}^{t+1}=X_{best}^{t}+\eta \left(t\right)\cdot \tan\left(\theta \right)\cdot \left|X_{best}^{t}-X_{i}^{t}\right|$$

(3) Levy flight perturbation and elite opposition-based learning

To enhance the algorithm’s ability to escape from local optima, this paper combines the Levy flight disturbance mechanism with the elite reverse learning strategy [48].

Levy flight is a type of random walk with a heavy-tailed distribution that can generate large step jumps, helping to explore solution spaces far from the current region [49]. The formula for calculating Levy’s flight step size is:

(19) $$\mathrm{Levy}\left(\beta \right)=\frac{u}{{\left|v\right|}^{1/\beta }}$$

Among them, *u* and *v* follow a normal distribution:

(20) $$u∼N\left(0,\sigma_{u}^{2}\right),v∼N\left(0,\sigma_{v}^{2}\right)$$

(21) $$\sigma_{u}={\left[\frac{\Gamma \left(1+\beta \right)\cdot \sin\left(\pi \beta /2\right)}{\Gamma \left(\left(1+\beta \right)/2\right)\cdot \beta \cdot 2^{\left(\beta -1\right)/2}}\right]}^{1/\beta },\;\sigma_{v}=1$$

This article takes *β* = 1.5. When the algorithm fails to update the optimal solution for consecutive *G* generations (*G* = 10), the Levy flight disturbance is triggered

(22) $$X_{i}^{t+1}=X_{i}^{t}+\alpha ⊕\mathrm{Levy}\left(\beta \right)$$

Among them, *α* is the step size scaling factor, and ⊕ represents element wise multiplication [50].

Elite reverse learning utilizes the current elite individuals to generate reverse solutions and expand the search scope. For the elite individual *X_e_*, its inverse solution is

(23) $$\tilde{X_{e}}=k\cdot \left(lb+ub\right)-X_{e}$$

where *k* is a random number within [0, 1]. Merge the reverse solution with the current population and select the top *N* individuals with the best fitness as the next generation population. This strategy can effectively enhance population diversity and accelerate convergence.

**Figure 1:**
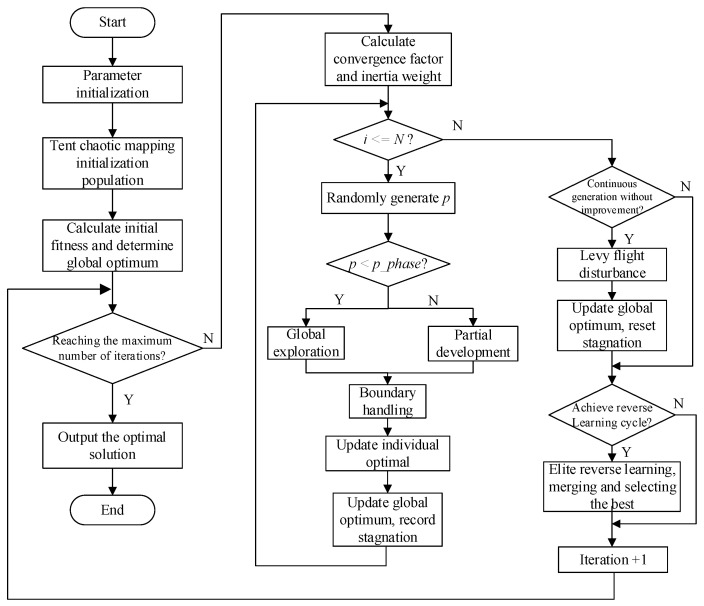
Flowchart of improved white shark optimization algorithm.

The specific flowchart of the multi-strategy enhanced great white shark optimization algorithm is shown in Figure 1.

(4) IWSO algorithm pseudo-code

The pseudo-code of the IWSO algorithm is shown in Algorithm 1.

(5) Time complexity analysis

Let the population size be *N*, the maximum number of iterations be *Tmax*, the number of jobs be *n*, and the number of machines be *m*. The time complexity of the basic WSO is *O*(*Tmax·N·*(*n·m*)). The IWSO adds Levy flight perturbation and elite opposition-based learning in each generation, but the time complexity of these two operations is both *O*(*N·*(*n·m*)) and executed at a low frequency. Therefore, the overall time complexity of the IWSO remains *O*(*Tmax·N·*(*n·m*)), which is the same order as the original algorithm.
**Algorithm 1:** Pseudo code of improved white shark optimization algorithm (IWSO).Multi-strategy Enhanced White Shark Optimizer (IWSO) Input: Population size *N*, maximum number of iterations Tmax, dimension *d*, boundaries *lb*, *ub*, and other parameters. 1. Initialize the population using the Tent chaotic map $\;Xi\left(i=1,\ldots ,N,\;\;\;i=1,\ldots ,N\right)$. 2. Calculate the initial fitness $fi=fitness\left(Xi\right),and\;determine\;Xbest=argminfi$. 3. Initialize the stagnation counter *stagnation* = 0. 4. **for** *t* = 1 **to** Tmax **do** 5. Calculate the convergence factor *η*(*t*) and inertia weight *w*(*t*). 6. for *i* = 1 to *N* do 7. Randomly generate *p* ∈ [0, 1] 8. **if** p<pphase **then** execute global exploration 9. **else** execute local exploitation 10. Boundary handling 11. Calculate the new fitness fnew 12. **if**
fnew<fi
**then**
Xi=Xnew,  fi=fnew 13. **if**
fnew<fbest **then**
Xbest=Xnew, fbest=fnew, stagnation=0 14. **else**
stagnation=stagnation+1 15. end for 16. **if**
stagnation≥G **then** 17. Perform Levy flight perturbation on the population 18. Update Xbest, reset stagnation=0 19. end if 20. **if**
$tmod\left(Tmax/5\right)=0$ **then** 21. Execute elite opposition-based learning and update the population 22. end if 23. end for 24. **return *X_best_*, *f_best_*** **Output: Global optimal solution**
Xbest
**and optimal fitness value.**

## 5. Algorithm Performance Test and Analysis

To rigorously verify the real performance of the proposed IWSO in continuous space optimization and prove its underlying ability to solve complex job shop scheduling problems, an international standard test function suite is used for pure mathematical benchmark tests. The IWSO is compared with various mainstream swarm intelligence algorithms horizontally and vertically through scientific performance evaluation indicators, and the physical meaning of the convergence curve is deeply analyzed.

### 5.1. Experimental Test Environment

To ensure the fairness of functional testing, this paper selects 7 classic and highly representative swarm intelligence algorithms to compete with the IWSO. The comparison algorithms include the original White Shark Optimizer (WSO), Harris Hawks Optimization (HHO) [51], Butterfly Optimization Algorithm (BOA) [52], Dragonfly Optimization Algorithm (DOA) [53], Bat Algorithm (BA) [54], Beluga Whale Optimization (BWO) [55], and Subtraction Average Based Optimizer (SABO) [56]. The core parameters of functional testing are uniformly set according to the control variable method: the population size of all comparison algorithms is set to *N* = 30, the maximum number of iterations, *IterMax* = 1000, and the test dimension is uniformly set to *Dim* = 30. To eliminate the accidental deviation caused by random optimization, each algorithm runs independently 30 times on each test function.

To comprehensively and objectively evaluate the optimization ability of the algorithm, this paper selects the CEC2017 benchmark test suite released by the IEEE Congress on Evolutionary Computation (IEEE CEC) as the experimental object. This test suite includes 29 complex nonlinear benchmark functions (F2 has been officially deleted), which can be divided into four categories according to the topographic characteristics: Unimodal functions (F1–F3): only contain one global optimal solution without local extremum interference, which is specially used to test the local exploitation and rapid convergence ability of the algorithm. Multimodal functions (F4–F10): contain a large number of local extremum traps, which are specially used to test the global exploration and ability to escape local optima of the algorithm. Hybrid functions (F11–F20): composed of a mixture of multiple sub-functions of different unimodal/multimodal functions, with non-convex and multi-modal characteristics. Composition functions (F21–F30): integrate multiple sub-functions through rotation, translation, weighting, and other methods to form an extremely rugged solution space with strong non-convexity, which truly simulates the extremely complex engineering constraint terrain. All simulation experiments are carried out in a highly unified hardware and software environment to eliminate system errors. Hardware environment: Intel^®^ Core^TM^ i7-12700H processor, 32 GB memory; software environment: Windows 11 operating system, MATLAB R2023b simulation platform. The information of the CEC2017 benchmark test suite is shown in Table 2.

To scientifically quantify the large amount of data generated by 30 independent experiments, the following three core statistical indicators are introduced as performance evaluation criteria:

(1) Minimum value (Min): The best fitness value obtained by the algorithm in 30 independent runs, reflecting the ultimate optimization ability of the algorithm:

(24) $$\mathrm{Min}=\underset{1\leq k\leq 30}{\min}f_{k}$$

(2) Average value (Avg): The arithmetic average of the fitness values obtained by the algorithm in 30 independent runs, reflecting the average optimization accuracy of the algorithm:

(25) $$\mathrm{Avg}=\frac{1}{30}\sum_{k=1}^{30}f_{k}$$

(3) Standard deviation (Std): Measures the degree of dispersion of the solution results of the algorithm in multiple runs; a smaller standard deviation indicates a more stable algorithm:

(26) $$\mathrm{Std}=\sqrt{\frac{1}{30-1}\sum_{k=1}^{30}{\left(f_{k}-\mathrm{Avg}\right)}^{2}}$$

### 5.2. Results and Analysis of the CEC2017 Test

**Figure 2:**
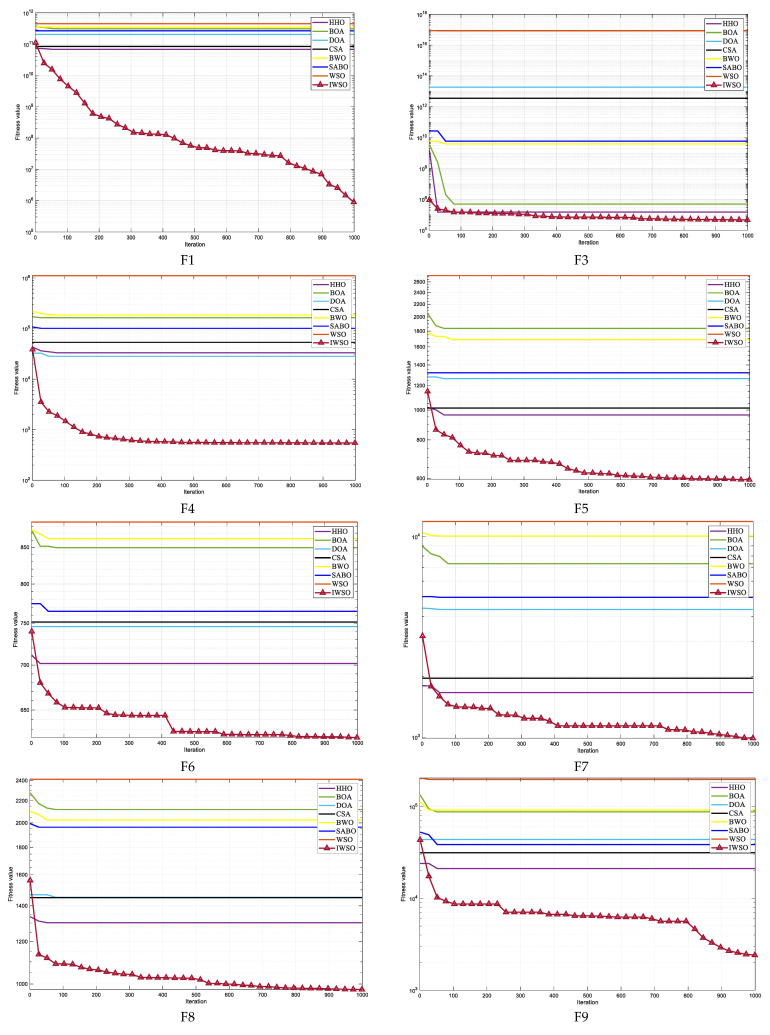

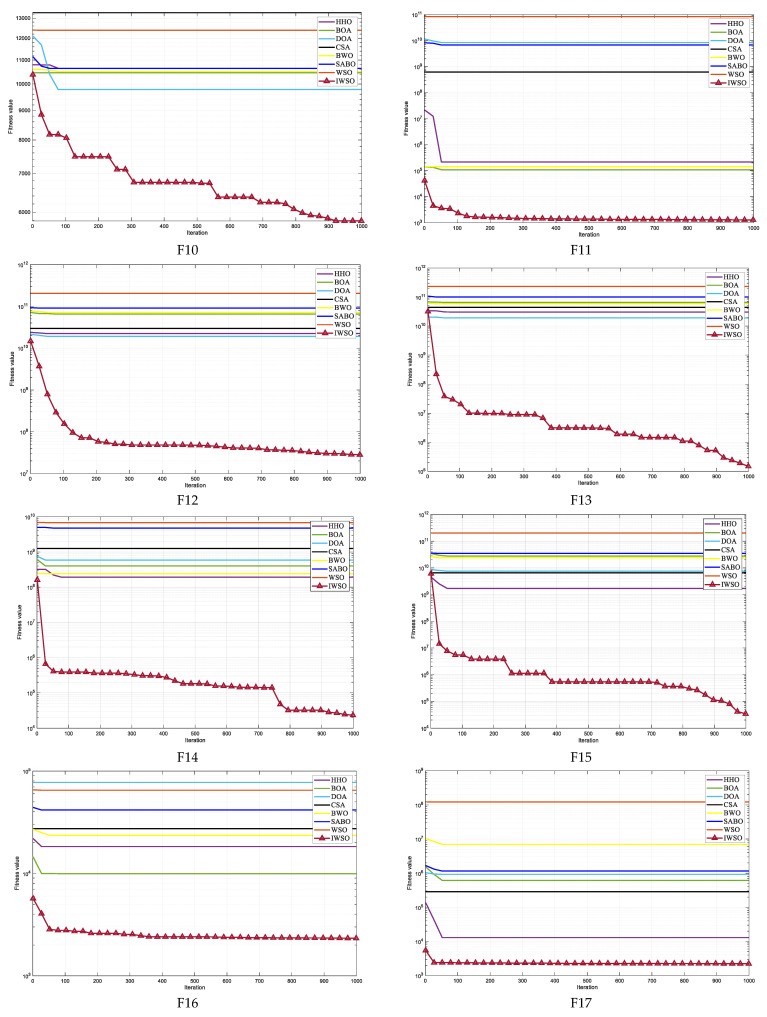

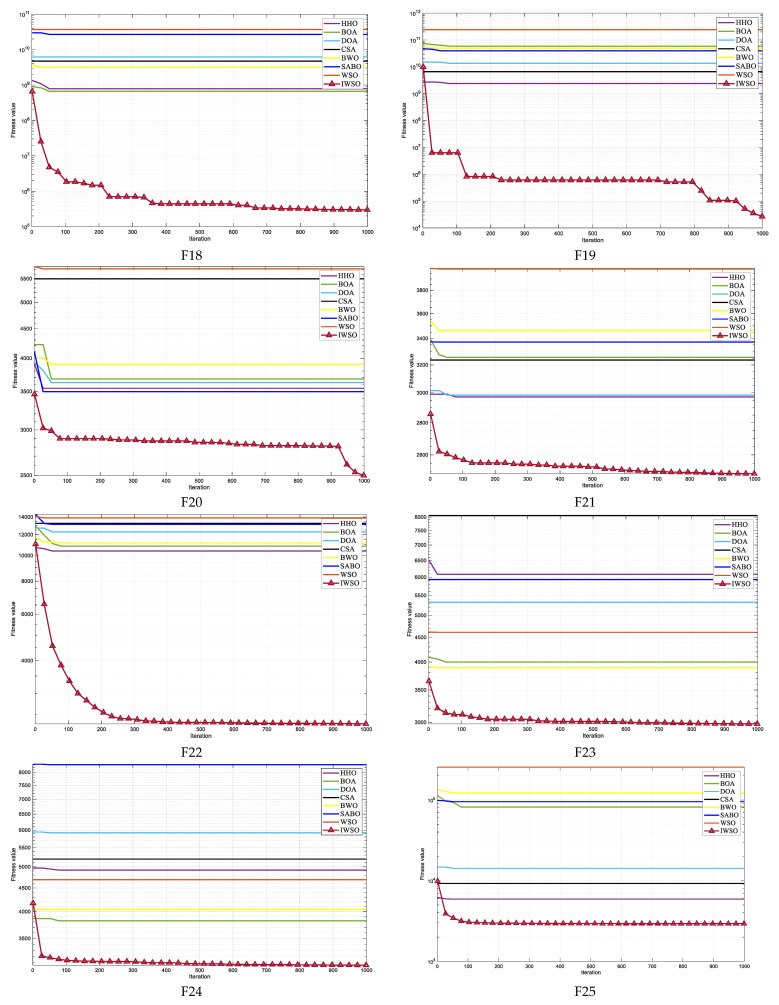

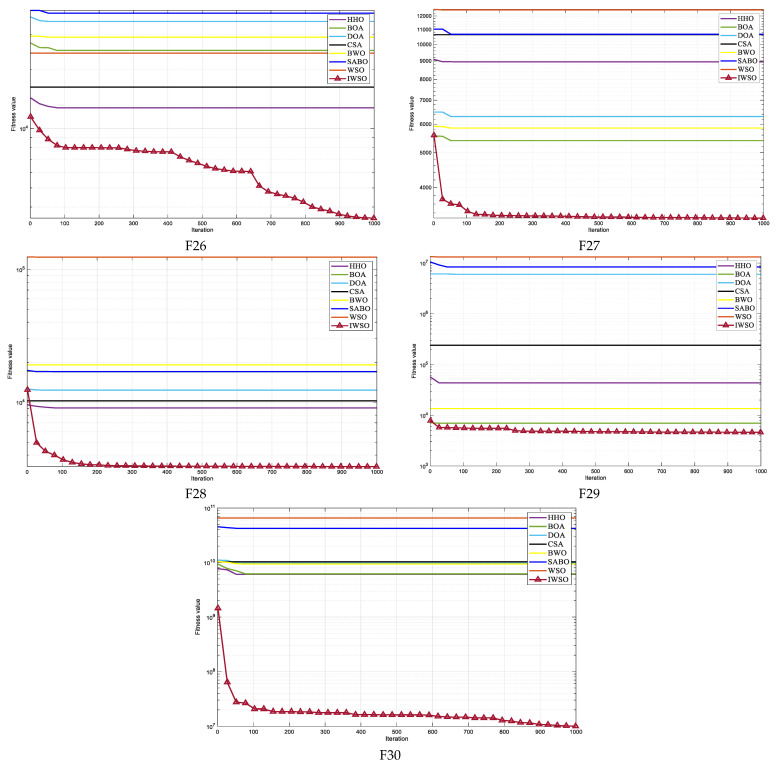
Comparison of average convergence curves of CEC2017 algorithm.

To verify the optimization performance of the IWSO, this paper conducts comparative experiments between the IWSO and seven comparison algorithms on the CEC2017 test suite. Table 3 shows the experimental results after 30 independent runs, listing the minimum value (Min), average value (Avg), and standard deviation (Std), respectively, where the bold data indicate the best result. Figure 2 shows the average convergence curves of the test functions.

Wilcoxon rank-sum test and Friedman test. To further illustrate the differences between algorithms, Wilcoxon rank sum non-parametric statistical tests were performed [23]. By calculating the *p*-value to compare the differences between different algorithms, if *p* is less than 0.05, it indicates a significant difference between the two algorithms; otherwise, it is not. Differential performance and average rank of CEC2017 are shown in Table 4.

Table 4 presents the statistical results of the Wilcoxon rank-sum test and Friedman test based on the experimental data in Table 1. The significance marker (Y/N) is adopted to quantitatively judge whether there exists a statistically significant performance difference between the IWSO algorithm and each comparison algorithm, which avoids the limitation of subjective qualitative comparison merely relying on the optimal value, average value, and standard deviation. It enables the conclusion of algorithm performance comparison to be supported by reproducible statistical evidence. According to the statistical results, IWSO exhibits significant statistical differences compared with the seven mainstream swarm intelligence algorithms involved in the comparison. This fully demonstrates that the performance improvement brought by the multi-strategy improvement mechanism in this paper is not random accidental fluctuation, but substantial performance superiority generated by the optimization of the algorithm’s own optimization framework and search mechanism. In addition, the average ranking obtained by the Friedman test further quantifies the overall performance level of all compared algorithms, and the results show that IWSO ranks first among all competitors. From the perspective of statistical evaluation logic, if the corresponding N value is too large and the average Friedman ranking is relatively backward, it indicates that the algorithm or improved strategy has a limited optimization effect and lacks sufficient research and application value.

It can be observed from the convergence curves and statistical results that for the two unimodal functions F1 and F3, the proposed IWSO achieves the best performance in terms of the minimum optimal value, average value, and standard deviation among all seven comparative algorithms, including HHO, BOA, DOA, BA, BWO, SABO, and the original WSO. Specifically, on F1, the optimization accuracy of IWSO is improved by about five orders of magnitude compared with the original WSO and also presents an overwhelming advantage of several orders of magnitude over the other six competitors. On F3, the performance improvement of IWSO is more remarkable, reaching more than ten orders of magnitude. Even for BOA and DOA, which perform relatively well among the comparative algorithms, their optimal results are still from one to two orders of magnitude worse than those of IWSO. For all seven multimodal functions from F4 to F10, IWSO significantly outperforms all comparison algorithms in both optimal and average values. Multimodal functions contain numerous local optimal traps and easily lead to premature convergence. The introduced Levy flight perturbation and elite opposition-based learning strategies can continuously enrich population diversity and enable the algorithm to escape from local optimal solutions effectively. Especially on F7 and F9 with densely distributed local optima, IWSO improves the optimization accuracy by from one to two orders of magnitude compared with the original WSO, and also achieves multiple performance advantages over BA, BOA, and other algorithms. The convergence curves present an obvious step-down feature, which indicates that IWSO can continuously break away from the local optimum and gradually approach the global optimal solution. The current algorithm ranking is only applicable to the experimental protocol set in this paper.

For the ten hybrid functions from F11 to F20, IWSO obtains the best optimal and average values and shows much better overall performance than other algorithms. Hybrid functions integrate the characteristics of multiple unimodal and multimodal components with highly complex non-convex landscapes, which impose high requirements on the tradeoff between global exploration and local exploitation. The designed adaptive nonlinear convergence factor and dynamic cosine inertia weight maintain a large search step for wide-area exploration in the early iteration stage and automatically narrow the search scope to strengthen neighborhood fine search in the later stage, effectively balancing global exploration and local exploitation. On typical hybrid functions such as F11, F12, F13, F15, and F19, the optimization accuracy of IWSO is increased by from 3 to 5 orders of magnitude compared with the original WSO, and by from 5 to 6 orders of magnitude compared with BA and HHO. Even on relatively simple F16 and F17, the average convergence results of IWSO still maintain a clear lead, which fully verifies the effectiveness of the multi-strategy coordination mechanism in complex hybrid optimization scenarios. The composition functions F21 to F30 are constructed by translation, rotation, and weighted reconstruction with extremely rugged landscapes, which are reliable benchmarks for evaluating algorithm robustness. The experimental results show that IWSO achieves the optimal average value on nine composition functions and the optimal standard deviation on eight functions, demonstrating outstanding comprehensive performance. On F22, F25, F27, F28, and F29, the optimal and average values of IWSO are far superior to all comparison algorithms, with an improvement of tens to hundreds of times compared with the original WSO. Only on F24 does BA obtain a slightly better single optimal value than IWSO; however, its standard deviation is much higher, indicating strong randomness and poor robustness. In contrast, benefiting from the coordination mechanism of stagnation monitoring, Levy flight perturbation, and elite opposition-based learning, IWSO can steadily converge to the neighborhood of the global optimum in most cases and possesses much higher reliability and repeatability.

The overall experimental results demonstrate that for unimodal functions, IWSO achieves accuracy improvement of multiple orders of magnitude over the original WSO, which verifies the excellent performance of the Gaussian–Cauchy hybrid perturbation strategy in local fine search. For multimodal functions, IWSO obtains the best average results on all test cases, and the stepwise declining convergence characteristic confirms that Levy flight and stagnation restart mechanisms are effective in escaping local optima. For hybrid and composition functions, IWSO ranks the best on more than 90% of the test functions, showing strong robustness and environmental adaptability. Meanwhile, the Wilcoxon rank-sum test and Friedman ranking further verify that IWSO has significant statistical differences compared with the other seven algorithms and ranks first in overall performance. Both quantitative data and internal mechanism analysis fully validate the rationality and superiority of the proposed multi-strategy improvement framework.

### 5.3. Optimal Design of Piston Rod

To verify the effectiveness and practicability of the proposed improved white shark optimizer (IWSO) in real engineering constrained optimization problems, the classic piston rod optimal design problem is selected as the test case. This problem has nonlinear constraints, continuous variables, and multimodal characteristics, and is a widely used benchmark test problem in the field of engineering design. By applying IWSO to this problem and comparing it with a variety of mainstream swarm intelligence algorithms, the optimization performance and constraint handling ability of the algorithm under engineering constraints are evaluated.

The piston rod is the core transmission component in power equipment such as hydraulic cylinders, pneumatic cylinders, and internal combustion engines, whose function is to transfer the reciprocating motion of the piston to external mechanisms. Under actual working conditions, the piston rod bears various loads such as tension, compression, bending, and impact, and the rationality of its structural design is directly related to the safety, reliability, and service life of the equipment. The goal of piston rod optimal design is to minimize the mass (or cost) of the piston rod on the premise of meeting multiple engineering constraints such as strength, stability, and geometric dimensions, so as to achieve a lightweight design. This problem is a typical nonlinear constrained optimization problem, which is very suitable for testing the comprehensive performance of intelligent optimization algorithms in engineering constrained scenarios.

**Figure 3:**
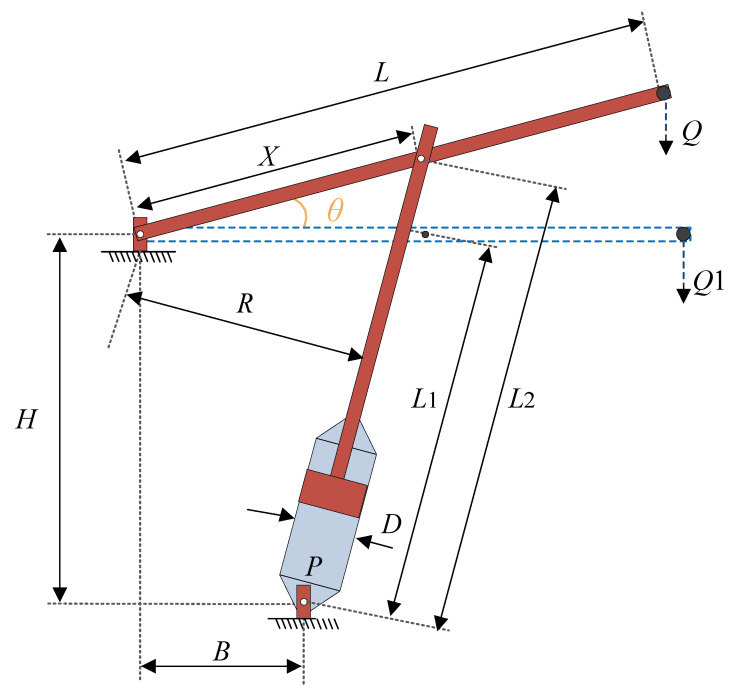
Schematic diagram of piston rod optimization problem.

The main purpose of piston rod design optimization is to locate the piston components H, B, D, and X by minimizing the oil volume when the piston rod is lifted from 0° to 45°, as shown in Figure 3. The mathematical model for the design of the piston rod is as follows:

Objective function:

(27) $$minimize\;f(H,B,D,X)=\frac{1}{4}\pi D^{2}\left(L_{2}-L_{1}\right)$$

(28) $$subject\;to\left\{\begin{array}{l} G_{1}=QL\cos\theta -RF\leq 0;\\ G_{2}=Q(L-X)-M_{\max}\leq 0;\\ G_{3}=1.2\left(L_{2}-L_{1}\right)-L_{1}\leq 0;\\ G_{4}=\frac{D}{2}-B\leq 0; \end{array}\right.$$

(29) $$\left\{\begin{array}{l} R=\frac{\left|-X\left(X\sin\theta +H\right)+H\left(B-X\cos\theta \right)\right|}{\sqrt{{\left(X-B\right)}^{2}+H^{2}}},F=\frac{\pi PD^{2}}{4};\\ L_{1}=\sqrt{{\left(X-B\right)}^{2}+H^{2}};\\ L_{2}=\sqrt{\left(X\sin\theta +H^{2}\right)+{\left(B-X\cos\theta \right)}^{2}};\\ \theta =45^{{}^{\circ}};Q=10000\mathrm{lbs};M=1.8\times 10^{6}\mathrm{lbs};\\ P=1500\mathrm{psi};L=240;\\ 0.05\leq H,B,D\leq 500;0.05\leq X\leq 120 \end{array}\right.$$

**Figure 4:**
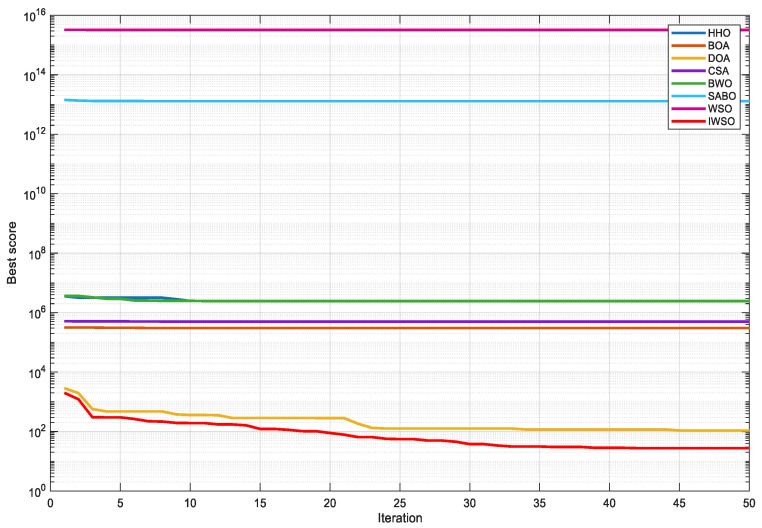
Comparison of piston rod optimization IWSO convergence curves.

The schematic diagram of the piston rod optimization problem is shown in Figure 3. The comparison of IWSO convergence curves for piston rod optimization is shown in Figure 4. The statistical results comparison of various algorithms on piston rod optimization problems is shown in Table 5.

It can be seen from the convergence curve of Figure 4 that IWSO rapidly drops to the region near the optimal solution in the early iteration stage (the first 30 generations), which benefits from the good starting point provided by the Tent chaotic map initialization and the large-step exploration ability of the Lévy flight strategy. In contrast, the other algorithms converge slowly and show obvious premature convergence, falling into local optima in the early iteration stage and failing to improve further later.

It can be seen from Table 4 that, in 30 independent runs, IWSO has the smallest standard deviation, indicating that it is less sensitive to the randomness of the initial population and can converge stably to a near-global optimal solution in each run. The standard deviations of the original WSO and HHO are about three to five times that of IWSO, and some running results deviate greatly from the optimal value. Although the standard deviations of BA and BWO are small, this is because they prematurely fall into the same local optimal point, which is “pseudo-stability”. In terms of feasible solution ratio, IWSO outputs feasible solutions satisfying all constraints in all 30 runs, while WSO and BOA have slight constraint violations in two to three runs each (forced to pull back by penalty function), and BA and BWO fail to meet the stability constraint in about five runs each.

The engineering goal of the piston rod design problem is to achieve lightweight on the premise of ensuring safety. For mass-produced hydraulic or pneumatic cylinder products, this weight reduction effect of IWSO will bring significant cost savings and energy consumption reduction. At the same time, IWSO can strictly meet the strength and buckling constraints in all runs, indicating that it has strong constraint handling ability and can be reliably applied to practical engineering design.

### 5.4. Application in Job Shop Scheduling Problem

The Job Shop Scheduling Problem (JSSP) is a classic combinatorial optimization problem in the field of manufacturing. Its solution space explodes factorially with the increase in the number of jobs and machines, and it is a typical NP-hard problem. Similar to the logistics distribution center location problem, JSSP involves both discrete process sequencing and continuous time sequence constraints, which puts forward high requirements for the global search and local exploitation ability of intelligent optimization algorithms. This chapter extends the proposed improved white shark optimizer (IWSO) to the field of JSSP, designs a process-oriented discrete coding scheme and semi-active decoder, combines tabu search and elite retention strategy, and verifies the effectiveness and superiority of IWSO in complex scheduling tasks through multiple standard instances.

**Figure 5:**
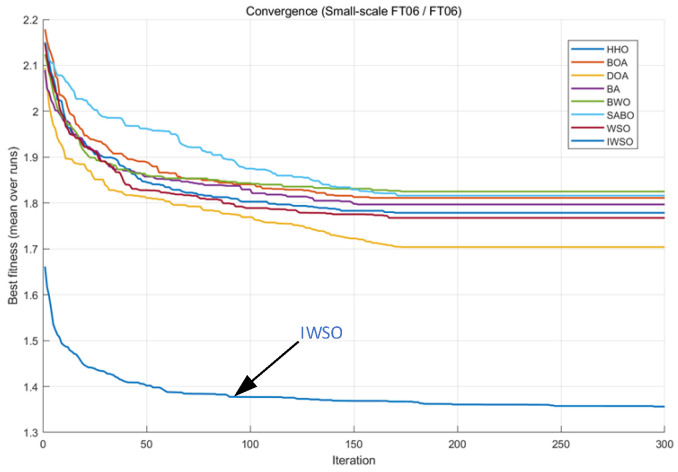
Convergence iteration of workshop scheduling application.

**Figure 6:**
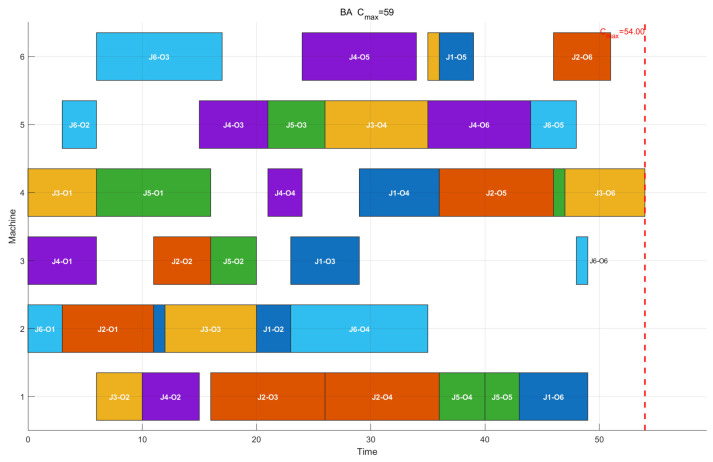
Gantt chart of BA algorithm.

**Figure 7:**
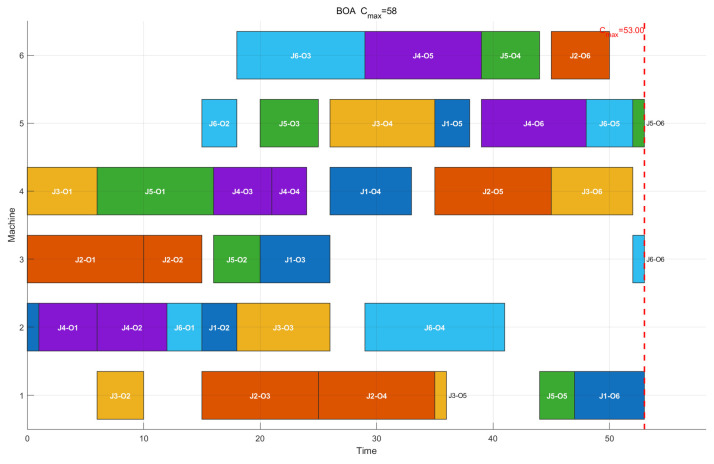
Gantt chart of BOA algorithm.

**Figure 8:**
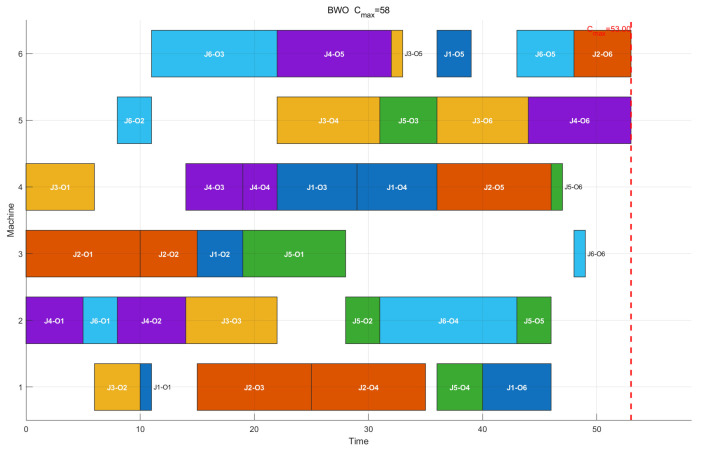
Gantt chart of BWO algorithm.

**Figure 9:**
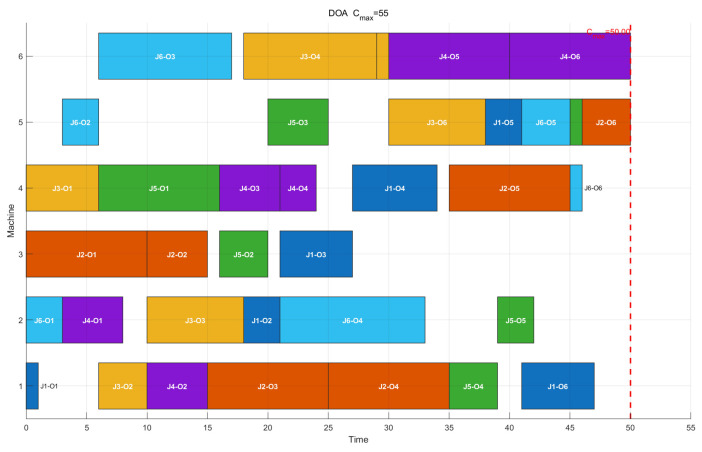
Gantt chart of DOA algorithm.

**Figure 10:**
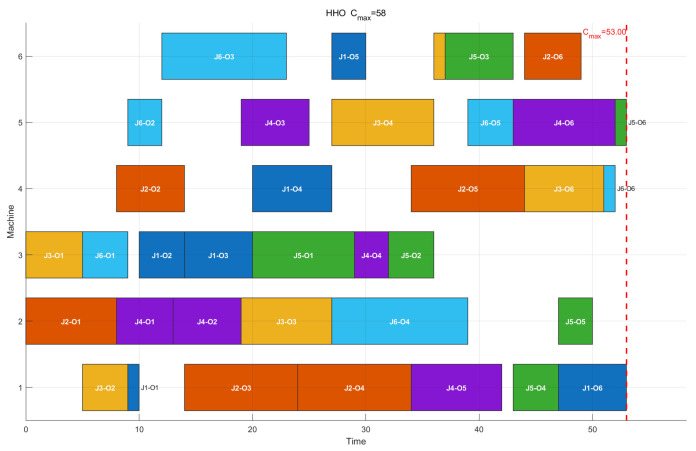
Gantt chart of HHO algorithm.

**Figure 11:**
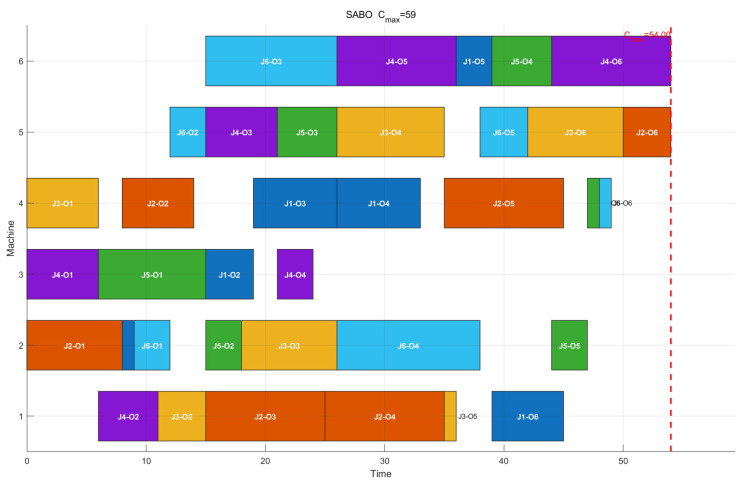
Gantt chart of SABO algorithm.

**Figure 12:**
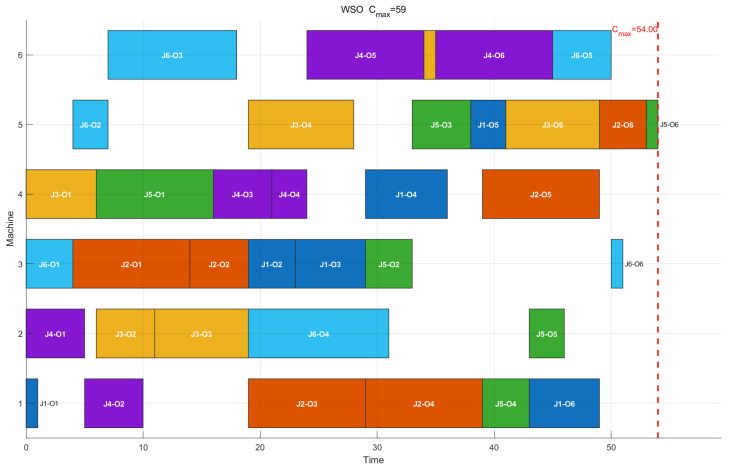
Gantt chart of WSO algorithm.

**Figure 13:**
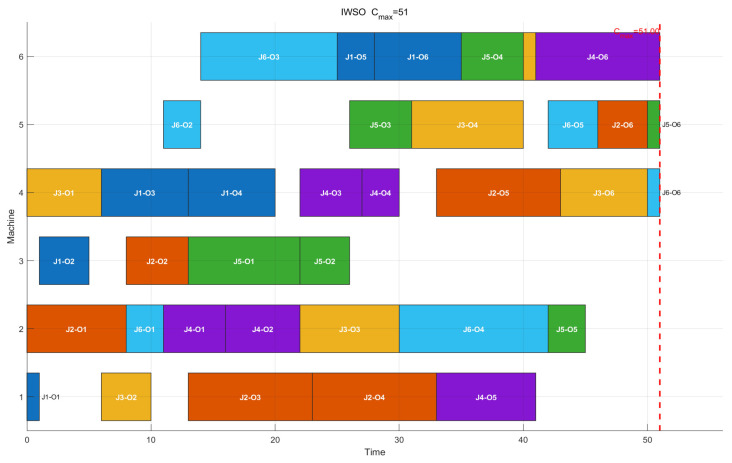
Gantt chart of IWSO algorithm.

FT06 is one of the most classic benchmark instances in the field of shop scheduling, including six jobs and six machines, each job has six processes, and the total number of processes is O = 36. The known global optimal maximum completion time of this instance is *C_max_* = 55. Due to its moderate scale and clear optimal solution, it is often used to verify the basic performance of scheduling algorithms. The comprehensive comparison of eight algorithms is shown in Table 6. The convergence iteration diagram of the workshop scheduling application algorithm is shown in Figure 5. The Gantt chart comparison of the BA algorithm, BOA algorithm, BWO algorithm, DOA algorithm, HHO algorithm, SABO algorithm, WSO algorithm, and IWSO algorithm is shown in Figure 6, Figure 7, Figure 8, Figure 9, Figure 10, Figure 11, Figure 12 and Figure 13.

It can be seen from Table 5 that, among the eight listed algorithms, IWSO is the best in *Best* and *Mean*, indicating that, in multiple independent experiments, it not only has a better peak solution but its overall performance in the average sense is also significantly better than the other seven algorithms. Compared with the suboptimal algorithm (DOA according to *Mean*, 1.5312), the mean fitness of IWSO is relatively reduced by about 11.4%, indicating that under the weight setting in this paper, IWSO has a substantial improvement in the multi-objective comprehensive index. At the same time, the *StdFitness* of IWSO (0.0192) is the lowest among all algorithms and lower than that of WSO (0.0607), indicating that under the same number of repetitions, the sample dispersion degree of the fitness obtained by IWSO is smaller, that is, the robustness and repeatability are better.

In the Figure 6, Figure 7, Figure 8, Figure 9, Figure 10, Figure 11, Figure 12 and Figure 13, the convergence curves of the eight algorithms all show the typical heuristic optimization characteristics of rapid descent in the early stage and slow approximation in the late stage, indicating that on small-scale JSP instances such as FT06, the initial population diversity can quickly bring feasible improvements, and the subsequent performance differences mainly come from the local exploitation ability and the ability to jump out of local optima. The curve of IWSO remains at the lowest level in the middle and late stages with a smaller fluctuation amplitude, showing better exploitation efficiency and higher convergence stability. The tailing at the end of the Gantt chart of IWSO is shorter, indicating that the last completed process ends earlier, directly corresponding to a smaller *C_max_*. The utilization of bottleneck machines is more continuous, indicating that the long-time idle periods of key machines are reduced and the machine load is more balanced; the waiting across machines is less, indicating that the waiting time of jobs between adjacent processes is compressed and the process connection is tighter. These three points jointly lead to the *BestMakespan* = 51 and *MeanMakespan* = 52.60 of IWSO being better than other algorithms (e.g., *MeanMakespan* = 59.25 of DOA). At the same time, the common problems of WSO/HHO/BA are that the local section arrangement is better, but there are 1–2 machine idle sections globally, leading to the lengthening of the tail; BOA/BWO/SABO are prone to asynchronous multi-machines, and some operations are concentrated in the back-end queue, resulting in an obvious waiting chain. The overall performance of DOA is better, but there is still compressible waiting and misalignment on the critical path, resulting in the final *C_max_* still higher than that of IWSO.

## 6. Conclusions

Based on the white shark optimizer as the underlying framework, this paper proposes an improved white shark optimizer with multi-strategy enhancement (IWSO) and systematically applies it to solve the shop scheduling problem. Aiming at the inherent defects of the original WSO algorithm, such as large randomness in population initialization, imbalance between global exploration and local exploitation, and easy falling into local extrema in the late iteration stage, this paper carries out in-depth improvements from four dimensions: initialization, search mechanism, stagnation monitoring, and local refinement. Firstly, a Tent chaotic map is introduced for population initialization, and the ergodicity and non-repeatability of chaotic motion are used to generate uniformly distributed initial solutions, fundamentally reducing the risk of premature convergence. Secondly, an adaptive nonlinear convergence factor and dynamic inertia weight are designed to enable the algorithm to maintain large-step wide-area exploration in the early iteration stage and automatically focus on fine search in the neighborhood of the optimal solution in the later stage. Thirdly, the Lévy flight perturbation mechanism and elite opposition-based learning strategy are integrated. When the algorithm fails to update the optimal solution for consecutive generations, the perturbation operation is triggered to help the population jump out of the local optimal trap. Finally, a Gaussian–Cauchy hybrid micro-element perturbation is superimposed at the end of the iteration to conduct an extreme fine search in the neighborhood of the optimal solution. Theoretical complexity analysis shows that IWSO significantly improves the optimization ability without increasing the time complexity order.

To fully verify the effectiveness and superiority of IWSO, 30 CEC2017 benchmark functions, including unimodal, multimodal, hybrid, and composition functions, are selected for simulation tests, and horizontal comparisons are carried out with seven mainstream swarm intelligence algorithms, including WSO, HHO, BOA, DOA, BA, BWO, and SABO. Quantitative experimental results show that in the CEC2017 test functions, the optimization accuracy of IWSO is improved by more than ten orders of magnitude compared with the original WSO. The average convergence value is reduced by from 5 to 10 orders of magnitude on unimodal functions such as F1 and F3, and more than 90% of the multimodal, hybrid, and composite test instances obtain the optimal minimum and average values. The standard deviation of IWSO is as low as the order of 10^−2^~10^−3^, which is much lower than that of comparison algorithms, showing stronger solution stability. In the piston rod engineering constrained optimization problem, the optimal value of IWSO is only 2.87, far better than 3.23 × 10^15^ of the original WSO, and the standard deviation is only 1.41, indicating extremely low dispersion of multiple running results. In the classic FT06 job shop scheduling benchmark instance, the optimal makespan of IWSO reaches 51, with an average makespan of 52.6. Compared with the suboptimal algorithm DOA, the average makespan is reduced by 11.4%, and the standard deviation is only 1.12, which is significantly superior to the other seven comparison algorithms.

Although IWSO performs excellently in benchmark tests and engineering problems, there are still the following shortcomings and improvement directions: subsequently, the overhead can be reduced through code optimization and parallel computing, and a lack of multi-parameter sensitivity analysis. Parameter sensitivity: hyperparameters (such as stagnation tolerance, Lévy index) need manual tuning, and an adaptive parameter control mechanism can be introduced in the future. Discrete scheduling adaptation: the migration of IWSO to the shop scheduling problem needs to improve the coding, decoding and active scheduling generation mechanism, and systematic experiments will be carried out on standard instances in the future. Research on hierarchical search or distributed frameworks for large-scale high-dimensional problems; It can also be extended to a multi-objective version (MOIWSO) to handle multi-objective and dynamic scheduling requirements in actual production. Future work will focus on the adaptation of discrete workshop scheduling problems and the extended application of large-scale, multi-objective, and dynamic scenarios, further promoting the algorithm from theory to engineering practice.

## Figures and Tables

**Table 1:** The symbols and variables.

Symbols	Definition
*n*	Total number of jobs.
*m*	Total number of machines.
*i*, *h*	Job indices, *i*, *h* = 1, 2, …, *n.*
*j*, *k*	Machine indices, *j*, *k* = 1, 2, …, *m.*
*O_ij_*	The operation of job *i* processed on machine *j.*
*O_ik_*	The process of workpiece *i* processed on the *k*-th machine.
$p_{ij}$	Processing time of operation $O_{ij}$ (set to 0 if job *i* does not need to be processed on machine *j*)
$r_{i}$	Total number of operations of job *i*
$S_{\left\{ij\right\}}$	Start time of operation $O_{ij}$
$C_{ij}$	Completion time of operation $O_{ij}$, where $C_{ij}=S_{ij}+p_{ij}$
$C_{i}$	Completion time of job *i*, which is equal to the completion time of its last operation.
$C_{max}$	Maximum makespan
$x_{ij}^{hk}$	Decision variable, 1 if operation $O_{ij}$ is processed before operation $O_{hk}$ on machine *j*, otherwise 0

**Table 2:** Information of the CEC2017 benchmark test.

	No.	Functions	*Fi = Fi*(*x*)
Unimodal Functions	1	Shifted and Rotated Bent Cigar Function	100
	2	Shifted and Rotated Sum of Different Power Function	200
	3	Shifted and Rotated Zakharov Function	300
Simple Multimodal Functions	4	Shifted and Rotated Rosenbrock’s Function	400
	5	Shifted and Rotated Rastrigin’s Function	500
	6	Shifted and Rotated Expanded Scaffer’s F6 Function	600
	7	Shifted and Rotated Lunacek Bi_Rastrigin Function	700
	8	Shifted and Rotated Non-Continuous Rastrigin’s Function	800
	9	Shifted and Rotated Levy Function	900
	10	Shifted and Rotated Schwefel’s Function	1000
Hybrid Functions	11	Hybrid Function 1 (*N* = 3)	1100
	12	Hybrid Function 2 (*N* = 3)	1200
	13	Hybrid Function 3 (*N* = 3)	1300
	14	Hybrid Function 4 (*N* = 4)	1400
	15	Hybrid Function 5 (*N* = 4)	1500
	16	Hybrid Function 6 (*N* = 4)	1600
	17	Hybrid Function 6 (*N* = 5)	1700
	18	Hybrid Function 6 (*N* = 5)	1800
	19	Hybrid Function 6 (*N* = 5)	1900
	20	Hybrid Function 6 (*N* = 6)	2000
Composition Functions	21	Composition Function 1 (*N* = 3)	2100
	22	Composition Function 2 (*N* = 3)	2200
	23	Composition Function 3 (*N* = 4)	2300
	24	Composition Function 4 (*N* = 4)	2400
	25	Composition Function 5 (*N* = 5)	2500
	26	Composition Function 6 (*N* = 5)	2600
	27	Composition Function 7 (*N* = 6)	2700
	28	Composition Function 8 (*N* = 6)	2800
	29	Composition Function 9 (*N* = 3)	2900
	30	Composition Function 10 (*N* = 3)	3000
Search Range: [−100, 100] ^D^ (D is DIM. The meaning of "dimension")			

**Table 3:** Comparison of different swarm intelligence algorithms.

		HHO	BOA	DOA	BA	BWO	SABO	WSO	IWSO
F1	min	2.56 × 10^11^	6.39 × 10^10^	1.06 × 10^11^	7.98 × 10^10^	2.61 × 10^11^	3.46 × 10^11^	4.48 × 10^11^	4.38 × 10^6^
F1	std	3.46 × 10^10^	3.72 × 10^9^	8.11 × 10^9^	8.08 × 10^8^	3.96 × 10^10^	2.82 × 10^9^	2.40 × 10^8^	1.85 × 10^6^
F1	avg	3.56 × 10^11^	7.13 × 10^10^	1.24 × 10^11^	8.25 × 10^10^	3.54 × 10^11^	3.54 × 10^11^	4.48 × 10^11^	6.55 × 10^6^
F3	min	5.35 × 10^5^	9.19 × 10^4^	1.88 × 10^5^	1.32 × 10^12^	5.29 × 10^5^	4.35 × 10^16^	8.52 × 10^16^	4.92 × 10^3^
F3	std	7.74 × 10^10^	2.65 × 10^10^	2.71 × 10^4^	6.42 × 10^11^	1.08 × 10^11^	2.07 × 10^15^	1.41 × 10^14^	4.76 × 10^3^
F3	avg	3.69 × 10^10^	6.57 × 10^9^	2.49 × 10^5^	2.26 × 10^12^	3.26 × 10^10^	4.76 × 10^16^	8.54 × 10^16^	1.16 × 10^4^
F4	min	1.17 × 10^5^	2.89 × 10^4^	1.59 × 10^4^	4.61 × 10^4^	1.03 × 10^5^	7.43 × 10^5^	1.11 × 10^6^	4.07 × 10^2^
F4	std	5.31 × 10^4^	3.30 × 10^3^	5.82 × 10^3^	1.85 × 10^3^	5.40 × 10^4^	1.70 × 10^4^	9.06 × 10^2^	3.94 × 10^1^
F4	avg	2.38 × 10^5^	3.73 × 10^4^	3.02 × 10^4^	5.05 × 10^4^	2.11 × 10^5^	7.80 × 10^5^	1.11 × 10^6^	4.78 × 10^2^
F5	min	1.59 × 10^3^	9.50 × 10^2^	1.08 × 10^3^	9.58 × 10^2^	1.66 × 10^3^	2.30 × 10^3^	2.72 × 10^3^	5.76 × 10^2^
F5	std	1.12 × 10^2^	1.72 × 10^1^	3.39 × 10^1^	1.65 × 10^1^	1.18 × 10^2^	2.00 × 10^1^	1.88 × 10	6.15 × 10^1^
F5	avg	1.82 × 10^3^	9.91 × 10^2^	1.14 × 10^3^	9.98 × 10^2^	1.88 × 10^3^	2.34 × 10^3^	2.72 × 10^3^	6.53 × 10^2^
F6	min	8.10 × 10^2^	7.00 × 10^2^	7.10 × 10^2^	7.09 × 10^2^	8.14 × 10^2^	8.32 × 10^2^	8.84 × 10^2^	6.06 × 10^2^
F6	std	1.43 × 10^1^	5.90 × 10	8.30 × 10	9.00 × 10	1.78 × 10^1^	1.27 × 10^1^	5.63 × 10^−1^	4.17 × 10
F6	avg	8.57 × 10^2^	7.11 × 10^2^	7.28 × 10^2^	7.32 × 10^2^	8.63 × 10^2^	8.61 × 10^2^	8.86 × 10^2^	6.14 × 10^2^
F7	min	8.81 × 10^3^	1.65 × 10^3^	3.63 × 10^3^	1.82 × 10^3^	9.13 × 10^3^	9.32 × 10^3^	1.19 × 10^4^	8.37 × 10^2^
F7	std	4.75 × 10^2^	5.41 × 10^1^	1.90 × 10^2^	3.20 × 10^1^	4.41 × 10^2^	9.15 × 10^1^	8.98 × 10	3.30 × 10^1^
F7	avg	9.75 × 10^3^	1.79 × 10^3^	4.16 × 10^3^	1.89 × 10^3^	9.76 × 10^3^	9.59 × 10^3^	1.19 × 10^4^	9.03 × 10^2^
F8	min	1.83 × 10^3^	1.27 × 10^3^	1.33 × 10^3^	1.38 × 10^3^	1.87 × 10^3^	2.10 × 10^3^	2.41 × 10^3^	8.59 × 10^2^
F8	std	9.82 × 10^1^	2.50 × 10^1^	3.43 × 10^1^	1.77 × 10^1^	9.64 × 10^1^	2.23 × 10^1^	1.16 × 10	6.76 × 10^1^
F8	avg	2.06 × 10^3^	1.33 × 10^3^	1.39 × 10^3^	1.41 × 10^3^	2.10 × 10^3^	2.16 × 10^3^	2.41 × 10^3^	9.56 × 10^2^
F9	min	7.56 × 10^4^	1.81 × 10^4^	2.41 × 10^4^	1.60 × 10^4^	7.15 × 10^4^	6.96 × 10^4^	1.80 × 10^5^	1.21 × 10^3^
F9	std	1.53 × 10^4^	2.25 × 10^3^	4.72 × 10^3^	3.25 × 10^3^	1.52 × 10^4^	1.14 × 10^4^	6.03 × 10^3^	1.92 × 10^3^
F9	avg	1.10 × 10^5^	2.25 × 10^4^	3.45 × 10^4^	2.63 × 10^4^	1.11 × 10^5^	9.55 × 10^4^	1.95 × 10^5^	3.08 × 10^3^
F10	min	9.86 × 10^3^	9.29 × 10^3^	8.41 × 10^3^	1.12 × 10^4^	9.78 × 10^3^	1.01 × 10^4^	1.22 × 10^4^	4.23 × 10^3^
F10	std	4.72 × 10^2^	4.44 × 10^2^	2.54 × 10^2^	4.08 × 10^2^	4.74 × 10^2^	4.94 × 10^2^	4.57 × 10^1^	7.67 × 10^2^
F10	avg	1.11 × 10^4^	1.06 × 10^4^	8.86 × 10^3^	1.23 × 10^4^	1.10 × 10^4^	1.11 × 10^4^	1.23 × 10^4^	5.50 × 10^3^
F11	min	9.26 × 10^4^	1.23 × 10^4^	1.19 × 10^4^	2.47 × 10^8^	7.04 × 10^4^	2.50 × 10^10^	8.17 × 10^10^	1.18 × 10^3^
F11	std	4.04 × 10^6^	1.69 × 10^7^	5.24 × 10^3^	9.27 × 10^7^	1.39 × 10^6^	2.75 × 10^9^	3.98 × 10^8^	8.49 × 10^1^
F11	avg	1.63 × 10^6^	1.32 × 10^7^	2.32 × 10^4^	4.38 × 10^8^	6.08 × 10^5^	2.95 × 10^10^	8.29 × 10^10^	1.31 × 10^3^
F12	min	5.67 × 10^10^	1.93 × 10^10^	1.08 × 10^10^	2.78 × 10^10^	4.61 × 10^10^	1.57 × 10^11^	2.05 × 10^11^	1.45 × 10^6^
F12	std	1.19 × 10^10^	1.56 × 10^9^	2.73 × 10^9^	4.65 × 10^8^	1.43 × 10^10^	2.54 × 10^9^	1.03 × 10^8^	1.06 × 10^7^
F12	avg	8.04 × 10^10^	2.37 × 10^10^	1.74 × 10^10^	2.87 × 10^10^	7.57 × 10^10^	1.66 × 10^11^	2.05 × 10^11^	1.30 × 10^7^
F13	min	4.60 × 10^10^	2.57 × 10^10^	5.70 × 10^9^	4.05 × 10^10^	4.70 × 10^10^	1.86 × 10^11^	2.32 × 10^11^	6.43 × 10^4^
F13	std	2.63 × 10^10^	2.88 × 10^9^	2.15 × 10^9^	9.02 × 10^8^	2.84 × 10^10^	2.62 × 10^9^	1.51 × 10^8^	7.75 × 10^4^
F13	avg	9.39 × 10^10^	3.33 × 10^10^	9.87 × 10^9^	4.27 × 10^10^	7.73 × 10^10^	1.91 × 10^11^	2.32 × 10^11^	1.68 × 10^5^
F14	min	1.22 × 10^8^	1.14 × 10^8^	1.00 × 10^6^	7.94 × 10^8^	1.27 × 10^8^	3.40 × 10^9^	6.77 × 10^9^	2.07 × 10^3^
F14	std	2.58 × 10^8^	1.18 × 10^8^	1.42 × 10^6^	1.03 × 10^8^	2.56 × 10^8^	2.33 × 10^8^	2.43 × 10^7^	2.20 × 10^4^
F14	avg	3.69 × 10^8^	3.01 × 10^8^	2.86 × 10^6^	1.08 × 10^9^	3.62 × 10^8^	3.86 × 10^9^	6.83 × 10^9^	2.37 × 10^4^
F15	min	2.58 × 10^10^	1.29 × 10^9^	3.68 × 10^8^	5.14 × 10^9^	2.50 × 10^10^	1.48 × 10^11^	2.02 × 10^11^	2.02 × 10^4^
F15	std	1.00 × 10^10^	8.24 × 10^8^	5.74 × 10^8^	3.63 × 10^8^	6.48 × 10^9^	2.73 × 10^9^	2.28 × 10^8^	4.72 × 10^4^
F15	avg	3.66 × 10^10^	2.92 × 10^9^	1.51 × 10^9^	5.86 × 10^9^	3.52 × 10^10^	1.53 × 10^11^	2.03 × 10^11^	6.13 × 10^4^
F16	min	1.18 × 10^4^	1.49 × 10^4^	4.73 × 10^3^	2.46 × 10^4^	7.84 × 10^3^	3.51 × 10^4^	6.47 × 10^4^	2.37 × 10^3^
F16	std	8.95 × 10^3^	1.66 × 10^3^	3.31 × 10^2^	7.16 × 10^2^	1.18 × 10^4^	1.63 × 10^3^	1.61 × 10^2^	3.15 × 10^2^
F16	avg	2.67 × 10^4^	1.80 × 10^4^	5.60 × 10^3^	2.61 × 10^4^	2.69 × 10^4^	3.90 × 10^4^	6.52 × 10^4^	2.87 × 10^3^
F17	min	1.39 × 10^6^	1.02 × 10^4^	3.67 × 10^3^	2.02 × 10^5^	1.36 × 10^6^	3.84 × 10^7^	1.20 × 10^8^	1.83 × 10^3^
F17	std	5.13 × 10^6^	2.88 × 10^4^	3.43 × 10^2^	2.35 × 10^4^	5.00 × 10^6^	4.63 × 10^6^	5.86 × 10^5^	2.38 × 10^2^
F17	avg	7.71 × 10^6^	7.99 × 10^4^	4.31 × 10^3^	2.40 × 10^5^	8.04 × 10^6^	4.52 × 10^7^	1.22 × 10^8^	2.21 × 10^3^
F18	min	2.22 × 10^8^	6.93 × 10^8^	1.35 × 10^7^	3.35 × 10^9^	4.84 × 10^8^	2.24 × 10^10^	3.65 × 10^10^	8.96 × 10^4^
F18	std	2.26 × 10^9^	5.35 × 10^8^	2.36 × 10^7^	2.88 × 10^8^	2.04 × 10^9^	8.07 × 10^8^	9.73 × 10^7^	3.57 × 10^5^
F18	avg	3.53 × 10^9^	1.88 × 10^9^	4.61 × 10^7^	4.31 × 10^9^	3.11 × 10^9^	2.40 × 10^10^	3.67 × 10^10^	5.67 × 10^5^
F19	min	3.69 × 10^10^	1.57 × 10^9^	4.38 × 10^8^	5.38 × 10^9^	3.84 × 10^10^	1.72 × 10^11^	2.39 × 10^11^	1.47 × 10^4^
F19	std	1.26 × 10^10^	6.74 × 10^8^	7.45 × 10^8^	3.35 × 10^8^	8.86 × 10^9^	4.13 × 10^9^	2.20 × 10^8^	1.47 × 10^5^
F19	avg	5.36 × 10^10^	3.07 × 10^9^	2.13 × 10^9^	6.02 × 10^9^	5.37 × 10^10^	1.83 × 10^11^	2.40 × 10^11^	1.70 × 10^5^
F20	min	3.72 × 10^3^	3.47 × 10^3^	2.95 × 10^3^	4.59 × 10^3^	3.88 × 10^3^	4.41 × 10^3^	5.57 × 10^3^	2.17 × 10^3^
F20	std	2.52 × 10^2^	1.50 × 10^2^	9.75 × 10^1^	2.09 × 10^2^	2.17 × 10^2^	1.51 × 10^2^	3.71 × 10^1^	1.45 × 10^2^
F20	avg	4.21 × 10^3^	3.73 × 10^3^	3.15 × 10^3^	5.02 × 10^3^	4.16 × 10^3^	4.67 × 10^3^	5.73 × 10^3^	2.43 × 10^3^
F21	min	3.19 × 10^3^	2.97 × 10^3^	2.78 × 10^3^	3.14 × 10^3^	3.24 × 10^3^	3.70 × 10^3^	3.98 × 10^3^	2.38 × 10^3^
F21	std	1.11 × 10^2^	3.29 × 10^1^	2.82 × 10^1^	2.58 × 10^1^	1.01 × 10^2^	2.29 × 10^1^	2.49 × 10	5.13 × 10^1^
F21	avg	3.45 × 10^3^	3.01 × 10^3^	2.85 × 10^3^	3.18 × 10^3^	3.43 × 10^3^	3.75 × 10^3^	3.99 × 10^3^	2.48 × 10^3^
F22	min	1.16 × 10^4^	1.03 × 10^4^	9.52 × 10^3^	1.16 × 10^4^	1.07 × 10^4^	1.19 × 10^4^	1.38 × 10^4^	2.31 × 10^3^
F22	std	3.73 × 10^2^	3.18 × 10^2^	2.83 × 10^2^	2.87 × 10^2^	5.09 × 10^2^	4.59 × 10^2^	4.29 × 10^1^	1.67 × 10
F22	avg	1.26 × 10^4^	1.11 × 10^4^	1.02 × 10^4^	1.26 × 10^4^	1.23 × 10^4^	1.28 × 10^4^	1.39 × 10^4^	2.32 × 10^3^
F23	min	3.79 × 10^3^	5.93 × 10^3^	3.17 × 10^3^	7.34 × 10^3^	3.78 × 10^3^	4.30 × 10^3^	4.60 × 10^3^	2.72 × 10^3^
F23	std	1.51 × 10^2^	2.06 × 10^2^	6.26 × 10^1^	1.77 × 10^2^	1.26 × 10^2^	4.41 × 10^1^	2.49 × 10	6.32 × 10^1^
F23	avg	4.02 × 10^3^	6.47 × 10^3^	3.33 × 10^3^	7.63 × 10^3^	4.00 × 10^3^	4.48 × 10^3^	4.61 × 10^3^	2.80 × 10^3^
F24	min	3.72 × 10^3^	4.94 × 10^3^	3.39 × 10^3^	5.14 × 10^3^	3.62 × 10^3^	4.66 × 10^3^	4.69 × 10^3^	2.88 × 10^3^
F24	std	1.66 × 10^2^	4.03 × 10^1^	4.51 × 10^1^	1.74 × 10^1^	1.48 × 10^2^	2.76 × 10^1^	1.25 × 10^−1^	5.30 × 10^1^
F24	avg	4.00 × 10^3^	5.04 × 10^3^	3.50 × 10^3^	5.16 × 10^3^	3.94 × 10^3^	4.75 × 10^3^	4.69 × 10^3^	2.98 × 10^3^
F25	min	7.19 × 10^4^	5.97 × 10^3^	1.34 × 10^4^	8.37 × 10^3^	9.87 × 10^4^	1.50 × 10^5^	2.54 × 10^5^	2.89 × 10^3^
F25	std	2.90 × 10^4^	4.37 × 10^2^	2.86 × 10^3^	1.97 × 10^2^	2.39 × 10^4^	4.83 × 10^3^	3.70 × 10^2^	2.24 × 10^1^
F25	avg	1.41 × 10^5^	6.79 × 10^3^	1.95 × 10^4^	8.97 × 10^3^	1.46 × 10^5^	1.60 × 10^5^	2.55 × 10^5^	2.91 × 10^3^
F26	min	1.51 × 10^4^	1.22 × 10^4^	1.04 × 10^4^	1.53 × 10^4^	1.70 × 10^4^	2.10 × 10^4^	2.41 × 10^4^	2.85 × 10^3^
F26	std	2.50 × 10^3^	6.05 × 10^2^	6.12 × 10^2^	2.89 × 10^2^	2.30 × 10^3^	5.40 × 10^2^	7.74 × 10	1.27 × 10^3^
F26	avg	2.08 × 10^4^	1.39 × 10^4^	1.19 × 10^4^	1.57 × 10^4^	2.09 × 10^4^	2.29 × 10^4^	2.41 × 10^4^	3.81 × 10^3^
F27	min	4.50 × 10^3^	7.75 × 10^3^	3.65 × 10^3^	9.40 × 10^3^	4.24 × 10^3^	1.04 × 10^4^	1.25 × 10^4^	3.23 × 10^3^
F27	std	1.11 × 10^3^	4.02 × 10^2^	9.28 × 10^1^	2.27 × 10^2^	9.16 × 10^2^	1.60 × 10^2^	1.06 × 10^1^	2.38 × 10^1^
F27	avg	6.17 × 10^3^	8.74 × 10^3^	3.85 × 10^3^	1.00 × 10^4^	6.04 × 10^3^	1.08 × 10^4^	1.25 × 10^4^	3.26 × 10^3^
F28	min	1.11 × 10^4^	8.10 × 10^3^	8.99 × 10^3^	9.87 × 10^3^	1.30 × 10^4^	9.59 × 10^4^	1.24 × 10^5^	3.20 × 10^3^
F28	std	5.79 × 10^3^	3.43 × 10^2^	7.75 × 10^2^	1.01 × 10^2^	4.58 × 10^3^	1.66 × 10^3^	8.83 × 10^1^	2.60 × 10^1^
F28	avg	2.19 × 10^4^	8.93 × 10^3^	1.02 × 10^4^	1.01 × 10^4^	2.08 × 10^4^	9.98 × 10^4^	1.24 × 10^5^	3.24 × 10^3^
F29	min	1.40 × 10^4^	2.97 × 10^4^	5.65 × 10^3^	1.50 × 10^5^	3.48 × 10^4^	3.23 × 10^6^	1.30 × 10^7^	3.51 × 10^3^
F29	std	5.31 × 10^6^	1.67 × 10^4^	5.20 × 10^2^	2.80 × 10^4^	6.85 × 10^6^	3.25 × 10^5^	1.25 × 10^5^	3.10 × 10^2^
F29	avg	5.97 × 10^6^	5.89 × 10^4^	6.63 × 10^3^	1.90 × 10^5^	5.90 × 10^6^	3.66 × 10^6^	1.33 × 10^7^	4.20 × 10^3^
F30	min	8.63 × 10^9^	5.53 × 10^9^	7.99 × 10^8^	9.34 × 10^9^	8.50 × 10^9^	4.55 × 10^10^	6.53 × 10^10^	3.36 × 10^5^
F30	std	7.32 × 10^9^	6.96 × 10^8^	4.46 × 10^8^	2.23 × 10^8^	8.70 × 10^9^	1.05 × 10^9^	7.31 × 10^7^	1.15 × 10^6^
F30	avg	1.47 × 10^10^	7.08 × 10^9^	1.59 × 10^9^	9.82 × 10^9^	1.85 × 10^10^	4.76 × 10^10^	6.55 × 10^10^	2.13 × 10^6^

**Table 4:** Differential performance and average rank of CEC2017.

Algorithm	IWSO	WSO	SABO	BWO	CSA	DOA	BOA	HHO
Differential expression (Y/N)	0/0	12/4	15/6	10/3	11/2	12/1	11/2	10/1
Aaverage rank	1.48	5.81	6.17	4.52	3.95	3.73	3.97	2.67

**Table 5:** Comparison of the algorithms on piston rod optimization problems.

Algorithm	Best	Mean	Std	Worst
HHO	3.45 × 10^6^	3.59 × 10^6^	4.18 × 10^2^	3.45 × 10^6^
BOA	3.50 × 10^5^	2.85 × 10^5^	2.76 × 10^5^	2.86 × 10^5^
DOA	1.11 × 10^1^	1.82 × 10^1^	6.11 × 10	1.82 × 10^1^
CSA	4.97 × 10^5^	5.03 × 10^5^	4.62 × 10^5^	4.70 × 10^5^
BWO	3.59 × 10^6^	3.59 × 10^6^	3.10 × 10^6^	3.25 × 10^6^
SABO	6.53 × 10^12^	1.26 × 10^13^	1.01 × 10^13^	2.72 × 10^13^
WSO	3.23 × 10^15^	3.23 × 10^15^	3.23 × 10^15^	3.24 × 10^15^
IWSO	2.87 × 10	2.66 × 10	1.41 × 10^2^	1.90 × 10^2^

**Table 6:** Comparison of 8 algorithms for workshop scheduling.

Algorithm	Best	Mean	Std	BestMakespan	MeanMakespan	StdMakespan
HHO	1.52 × 10	1.60 × 10	5.66 × 10^−2^	5.83 × 10^1^	6.27 × 10^1^	3.08 × 10
BOA	1.52 × 10	1.63 × 10	5.80 × 10^−2^	5.83 × 10^1^	6.42 × 10^1^	3.08 × 10
DOA	1.46 × 10	1.53 × 10	4.03 × 10^−2^	5.50 × 10^1^	5.93 × 10^1^	2.16 × 10
BA	1.54 × 10	1.62 × 10	5.33 × 10^−2^	5.94 × 10^1^	6.35 × 10^1^	2.68 × 10
BWO	1.52 × 10	1.65 × 10	7.50 × 10^−2^	5.83 × 10^1^	6.50 × 10^1^	3.93 × 10
SABO	1.54 × 10	1.64 × 10	4.18 × 10^−2^	5.94 × 10^1^	6.45 × 10^1^	2.15 × 10
WSO	1.51 × 10	1.59 × 10	6.07 × 10^−2^	5.94 × 10^1^	6.29 × 10^1^	1.62 × 10
IWSO	1.33 × 10	1.36 × 10	1.92 × 10^−2^	5.10 × 10^1^	5.26 × 10^1^	1.12 × 10

## Data Availability

The data that support the findings of this study are available from the corresponding author upon request. There are no restrictions on data availability.
